# How does adaptation sweep through the genome? Insights from long-term selection experiments

**DOI:** 10.1098/rspb.2012.0799

**Published:** 2012-10-25

**Authors:** Molly K. Burke

**Affiliations:** Department of Ecology and Evolutionary Biology, University of California, Irvine, CA 92697-2525, USA

**Keywords:** experimental evolution, genomics, selective sweeps

## Abstract

A major goal in evolutionary biology is to understand the origins and fates of adaptive mutations. Natural selection may act to increase the frequency of de novo beneficial mutations, or those already present in the population as standing genetic variation. These beneficial mutations may ultimately reach fixation in a population, or they may stop increasing in frequency once a particular phenotypic state has been achieved. It is not yet well understood how different features of population biology, and/or different environmental circumstances affect these adaptive processes. Experimental evolution is a promising technique for studying the dynamics of beneficial alleles, as populations evolving in the laboratory experience natural selection in a replicated, controlled manner. Whole-genome sequencing, regularly obtained over the course of sustained laboratory selection, could potentially reveal insights into the mutational dynamics that most likely occur in natural populations under similar circumstances. To date, only a few evolution experiments for which whole-genome data are available exist. This review describes results from these resequenced laboratory-selected populations, in systems with and without sexual recombination. In asexual systems, adaptation from new mutations can be studied, and results to date suggest that the complete, unimpeded fixation of these mutations is not always observed. In sexual systems, adaptation from standing genetic variation can be studied, and in the admittedly few examples we have, the complete fixation of standing variants is not always observed. To date, the relative frequency of adaptation from new mutations versus standing variation has not been tested using a single experimental system, but recent studies using *Caenorhabditis elegans* and *Saccharomyces cerevisiae* suggest that this a realistic future goal.

## 1. Selective sweeps: patterns for inferring process

What types of variation does natural selection act upon under different circumstances, and to what degree does selection erase this variation? In the classical view, selection acts upon newly arising beneficial mutations, but under some circumstances, standing genetic variation that was previously neutral or slightly deleterious may become the source for adaptive substitutions. In addition, though textbook models of adaptation predict that selection acts to increase the frequency of unconditionally beneficial alleles until they reach fixation in a population, this may not always be true. Attempts to resolve the origins and fates of adaptive mutations rely heavily upon the detection of so-called selective sweeps, which are evidenced by genomic patterns of reduced neutral variation at or near putatively causative sites. A classic or ‘hard’ selective sweep describes the process of a novel, major effect mutation arising on a single haplotype in a population and ultimately reaching fixation [1–3]. A ‘soft’ selective sweep, by contrast, describes the dynamics of selection acting on beneficial alleles present on many haplotypes in a population with standing genetic variation [4–7]. To clarify the admittedly obscure terminology, sweep patterns are hereafter classified as four specific types: (i) a complete hard sweep; (ii) an incomplete hard sweep; (iii) a complete soft sweep; and (iv) an incomplete soft sweep (table 1).

**Table 1.** Four potential selective sweep patterns that can be revealed by genomic sequence data. Embedded figures represent what a scan of heterozygosity or clonal diversity would illustrate, in terms of selective sweep signature near a causative site (location represented by the star).

sweep type	localized effect on heterozygosity		effect on haplotype diversity in replicate populations of a common selection treatment
hard sweep	footprint of zero heterozygosity	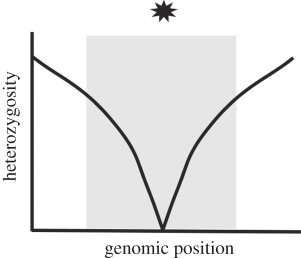	haplotype diversity among replicates is high. Unique de novo mutations on different genetic backgrounds fix in each replicate population.
incomplete hard sweep	local losses in heterozygosity that do not reach zero. Insufficient time has passed, or some unknown experimental parameter ‘stalls’ complete fixation.	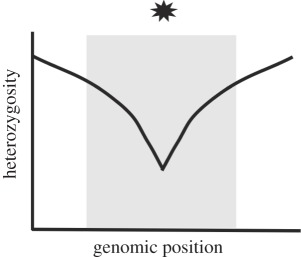	haplotype diversity is maintained (analogous to a complete hard sweep).
soft sweep	footprint of reduced heterozygosity narrower than that of a hard sweep. Footprint may not reach zero (e.g. dotted line) depending on the history of the causative variant.	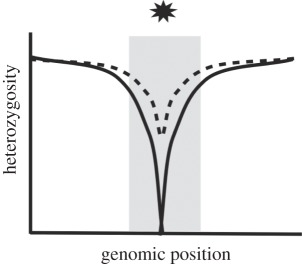	haplotype diversity among replicates is low. Replicate populations harbour the same low-frequency variants that reach fixation.
incomplete soft sweep	same as above, with a narrower footprint.	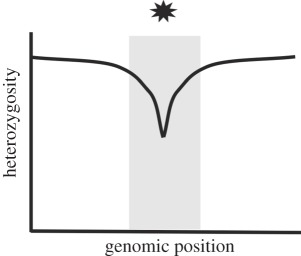	haplotype diversity is lost (analogous to a complete soft sweep).

In a complete hard sweep, a newly arising mutation reaches complete fixation and drags with it all linked neutral variation. Hard sweeps are fairly easy to detect; they leave a footprint of reduced heterozygosity (or clonal diversity in the case of haploid populations that undergo no recombination) roughly equal to the magnitude of one-tenth the selection on the allele in units of recombination [3]. In a complete hard sweep, heterozygosity should reach zero at or very near the causative site. In an incomplete hard sweep, zero heterozygosity is not achieved, for reasons that could include insufficient time-elapsed, epistatic interactions influencing sweep trajectory, or selection coefficients changing over time [8].

Soft sweeps will also leave a signature of reduced heterozygosity, but their detection is less straightforward. By definition, soft sweeps involve multiple copies of a selective allele contributing to a substitution, but these multiple copies may or may not be identical by descent [5–7]. When alleles are identical by descent, this is considered a single-origin soft sweep; selection acts on allelic variants already present at some frequency in the population. Cases where beneficial alleles enter the population independently, either through recurrent mutation or migration, are considered multiple-origin soft sweeps. An interesting recent example of adaptation via multiple-origin soft sweeps is provided by Karasov *et al.* [9]; they observed the same pesticide-resistance alleles arise independently on different haplotypes, and these alleles were selected upon simultaneously. A single-origin soft sweep should result in a narrower footprint of reduced heterozygosity than a hard sweep because the variant under selection is older and has been exposed to more recombination events with nearby neutral sites; when this type of sweep is complete, the footprint will reach zero. Multiple-origin soft sweeps, however, leave footprints of reduced heterozygosity that may not reach zero; as recurrent or migrant alleles enter the population on different haplotypes, neutral flanking variation is not necessarily swept away ([6,7]; reviewed in [10]). This situation is virtually indistinguishable from incomplete soft sweeps, where heterozygosity is expected to drop to non-zero levels because the variants under selection have not yet fixed.

Selective sweep patterns can therefore identify genomic regions where changes in allele frequencies correlate with phenotypic responses to selection, and they can also provide information about the origins and fates of alleles under selection. An important weakness of sweep analyses is that they yield no additional insights into the forces acting on adaptive alleles, such as epistasis, frequency-dependent selection or complex network interactions. Therefore, while they can be quite informative in ways, this review will discuss at length, it is important to note that sweep analyses are not a stand-alone method for understanding evolving populations.

## 2. What can evolution experiments reveal about mutational dynamics?

In model biological systems, experimental evolution is a versatile method that can test questions about the genetics underlying particular traits and the broader nature of adaptation. While historically, evolution experiments have been very successful at identifying candidate genes associated with phenotypes under direct selection, this is will not be discussed here (see [11], for review). Genomic data from evolution experiments, especially those that are highly replicated, have enormous potential to inform us about the origins and fates of adaptive mutations. Laboratory-selected populations can be screened for hard and soft selective sweeps, and this could potentially inform us about circumstances that lead to adaptation from de novo mutations versus standing genetic variation. It should also be possible to determine how often adaptive alleles reach complete fixation in the population, provided enough generations of selection are monitored. Ultimately, studying evolution experiments that have been designed with specific attention to population-genetic parameters may provide insights into how adaptation proceeds under certain environmental or demographic circumstances. As genomic studies of experimentally evolved populations are just beginning to accumulate, they provide some preliminary data on the origins and fates of adaptive mutations, but cannot yet fully address questions about populations in nature.

While the principles underlying experimental evolution technique are fairly unifying, studies use biological populations that differ in non-trivial ways. There is an important dichotomy between experimental evolution systems that use either asexually reproducing microbes or sexually reproducing higher eukaryotes. Microbial systems harbour some significant advantages, including fast generation times, the potential for large population sizes and large numbers of replicate populations [12,13], and small genome sizes that make genome resequencing easier. Microbial experimental evolutionists have the ability to dissect the genomics of their systems in sophisticated ways: they can identify new beneficial mutations when they occur in a population, estimate their effect on fitness and track their fates over thousands of generations. But in doing this, investigators are necessarily only documenting the dynamics of de novo beneficial mutations. While it is possible, though unusual, to start a microbial evolution experiment with an initially diverse population harbouring standing genetic variation, soft-sweep patterns can only be studied transiently. As soon as a beneficial variant becomes fixed, the entire haplotype bearing the variant sweeps to fixation as well, wiping out genetic variation and establishing a situation in which adaptation can only proceed via future new mutations. Therefore, in the absence of recombination, microbial evolution experiments are inadequate for assessing the degree to which natural selection acts on new mutations versus standing variation after the first fixation occurs in an evolving population.

Essentially, the opposite is true in an evolution experiment with sexual populations. Because these populations are freely recombining, there is the potential for both hard and soft sweeps to be detected. But in model sexual systems, most commonly *Drosophila*, populations cannot be handled practically at sizes much larger than 10^3^. This means that de novo beneficial mutations are very unlikely to arise in a given generation, and therefore standing genetic variation must drive adaptation in these experiments. In addition, generation times span weeks, meaning that a single investigator or laboratory group can only feasibly maintain a laboratory selection experiment for hundreds, rather than thousands, of generations. This means that even if one were to start with a sufficiently large population that de novo beneficial mutations occurred somewhat frequently, it would still be difficult to follow them through time to see whether they eventually completely fixed (though this is highly dependent on the size of the selection coefficient; see [14] for discussion).

This review will summarize the genomic data from long-term selection experiments in both asexual and sexual systems that bear on the dynamics of adaptive mutations (table 2). It will outline evidence, or lack thereof, for hard sweeps expected in microbial systems and soft sweeps expected in recombining systems. It will also discuss the limitations affecting particular selection experiments that prevent unambiguous inferences about these dynamics, and suggestions for how future selection experiments can be optimally designed.

**Table 2.** Outline of the key experiments featured in this review, highlighting various relevant parameters.

species	estimated *N*_e_	treatment	recombination?	initial standing genetic variation?	maximum number of generations	primary method of analysis	references
*S. cerevisiae*	10^9^	glucose limitation	no	no	500	yeast tiling array^a^	Kao & Sherlock [15]
*E. coli*	10^7^	glucose limitation	no	no	40 000	whole-genome sequencing^b^	Barrick & Lenski [16]
*S. cerevisiae*	10^5^ or 10^6^	bottleneck size	no	no	1000	sterility-marker assays	Lang *et al*. [12]
Bacteriophage (ID11)	10^4^ or 10^6^	bottleneck size	no	no	20	whole-genome sequencing^c^	Miller *et al.* [17]
*C. elegans*	500	complex environment; pathogen resistance	yes	no	50	fitness assays	Morran *et al*. [18]
*S. cerevisiae*	10^7^	temperature	initial rounds of recombination followed by asexual growth	yes	200	whole-genome sequencing^b^	Parts *et al.* [19]
*G. gallus domesticus*	35	body size	yes	yes	50	60 K chicken chip^b^	Johansson *et al.* [20]
*D. melanogaster*	10^3^	development time	yes	yes	600	whole-genome sequencing^b^	Burke *et al.* [14]
*D. melanogaster*	200	body size	yes	yes	100	whole-genome sequencing^b^	Turner *et al.* [21]
*D. melanogaster*	10^3^	hypoxia tolerance	yes	yes	200	whole-genome sequencing^b^	Zhou *et al.* [22]
*M. musculus domesticus*	80	body size	yes	yes	154	mouse diversity array^a^	Chan *et al.* [23]

^a^Affymetrix.

^b^Illumina.

^c^Sequetech.

## 3. In selection experiments with microbes, incomplete sweeps are observed more often than predicted

In most microbial evolution experiments, there is no exchange of genetic material between organisms, and in these cases where recombination is absent, the entire genome can be considered completely linked. The classical model of adaptive evolution in an asexual population proposes that each adaptive mutation occurs in succession. When a beneficial mutation arises, the entire clonal haplotype bearing that mutation experiences a complete hard selective sweep, and each adaptive clone in an evolving population is derived from the one preceding it [24]. This model of evolution by complete clonal replacement has been the dominant paradigm in microbial population genetics since the mid-twentieth century [25,26], and early *Escherichia coli* and *Saccharomyces cerevisiae* evolution experiments provided support for this theory [26–28]. While historically, evolution experiments have focused on the functional biology of the ‘winning’ clones [29,30], within the last few years whole-genome resequencing technologies have made it possible to more clearly characterize the dynamics of molecular changes underlying adaptive events over an evolutionary trajectory. All of the studies described in this section are non-recombining microbial evolution experiments that are initiated with isogenic, clonal populations (§6 discusses a single microbial evolution experiment where this is not the case). As such, they necessarily cannot inform us about how adaptation may proceed via standing genetic variation; hard sweeps are the only type that can occur in these populations. Interestingly, increasing genomic data suggest that *complete* hard sweeps via clonal replacement may more often be the exception than the rule.

Clonal haplotype diversity can be generated quickly in a microbial population when multiple de novo mutations arise at the same time. Haplotypes that carry different beneficial mutations compete with one another and thereby interfere with each other's spread and substitution (reviewed in [31]). This clonal interference, demonstrated in evolving populations of viruses [32], bacteria [33] and yeast [15], dominates the dynamics of microbial populations, and results in transient levels of haplotype diversity which slow the time it takes to fix the haplotype bearing the most beneficial mutation. Clonal interference dynamics assume that all but one lineage will eventually be excluded by the clone with the most beneficial mutation or combination of mutations (a genome-wide complete hard sweep). But the idea of complex clonal interference, whereby clonal haplotypes do not fix, or do not fix in a predictable way, has continued to garner empirical support. Kao & Sherlock [15] seeded replicated chemostat cultures with equal numbers of three fluorescently labelled, otherwise isogenic haploid yeast strains and monitored their growth in a glucose-limited environment. While they observed the expansion and contraction of subpopulations, this rarely resulted in complete clonal replacement, over a timescale of approximately 500 generations. In a much longer evolution experiment with *E. coli*, analyses of the genomic data suggest that complete clonal replacement is only one of several possible outcomes. Barrick & Lenski [16] identified different trajectories of clonal lineages at various timepoints over the course of 40 000 generations of evolution in minimal media. The most common trajectory was a complete hard sweep; the authors estimate that about 50 per cent of the beneficial mutations that arose in the experimental populations eventually fixed. Less common, but significant alternative trajectories included beneficial mutations that were lost owing to the fixation of a completing clone, and beneficial mutations that remained transient in the population; the authors suggest that these examples of incomplete clonal replacement could represent frequency-dependent adaptations.

Data from a recent *S. cerevisiae* study also provides evidence for incomplete hard sweeps. Lang *et al.* [12] used a sterility-advantage marker genotype (cf*.* [34]) to set up an elegant 1000-generation evolution experiment whereby marker mutations would have one of two selection advantages (*s* = 0.6% and *s* = 1.5%), and populations were of two sizes (*N*_e_ = 10^5^ and *N*_e_ = 10^6^). Once marker mutations arose, they experienced four general fates. The simplest case was a complete hard sweep: a spontaneous sterile mutation arose and increased in frequency until it fixed; these were not common, only observed in the small population size treatment, and only when the larger selective advantage was conferred. They also observed cases in which the mutations rose to intermediate frequency and remained there for hundreds of generations, suggesting the action of frequency-dependent selection. Most commonly, the beneficial mutations rose to some frequency, but were outcompeted by a more-fit lineage before they were able to fix, and this can be considered standard clonal interference. More complicated trajectories were also observed, in which clonal interference stopped a mutation's initial increase in frequency, but only for a short time before the mutation increased in frequency again. This could reflect a second beneficial mutation occurring in a declining, competing clone. This has generally been termed the multiple-mutation model of clonal interference [35]. Miller *et al.* [17] experimentally evolved phage at two bottleneck sizes (*N*_e_ = 10^4^ and *N*_e_ = 10^6^). After 20 passages, they observed that smaller treatments sometimes experienced complete hard sweeps, while the large treatments experienced very complex clonal interference dynamics involving multiple mutations.

These recent empirical data suggest that even in the simplest asexual experimental evolution constructs (i.e. only one substitution can be selected on at once; there is no standing genetic variation; populations are large enough that beneficial mutations are not rare), the dynamics of adaptation are complex. Textbook models of adaptation assume that such populations should evolve via the complete fixation of adaptive mutations, but recent empirical data repeatedly show that this does not necessarily happen. The reasoning behind why incomplete hard sweeps are often observed in these experiments remains largely speculative. But it is clear that even in initially clonal populations, new mutations can generate variation rapidly enough to strongly influence the dynamics of adaptation.

## 4. Selection experiments with sexual species in theory allow both sweep types, but in practice do not

Standing genetic variation cannot drive ongoing adaptation in an evolving microbial population because as soon as an initial sweep occurs, it wipes out any variation generated by clonal interference. Any future sweeps will be detected as hard sweeps for this reason; fixation in populations without recombination leads to population-wide clonality, even if this clonality is transient. On the other hand, sexual recombination allows for a mutation to fix, without genome-wide elimination of genetic variation. Neutral variation linked to the causal site will experience a sweep, while the rest of the genome remains unaffected. Therefore, repeated whole-genome resequencing from evolving sexually reproducing populations can in theory reveal whether substitutions are generally driven by new mutations or standing genetic variation. In practice though, this is flawed. It is well known that most quantitative traits respond quickly to laboratory or artificial selection (cf. [36]), and in these experiments, there is almost no time for new mutations to occur. In addition, evolution experiments using sexual systems do not easily accommodate large population sizes (e.g. *Drosophila* populations tend to have long-term *N*_e_ < 10^3^), and this makes the probability of a beneficial de novo mutation arising in a single generation rare. It is therefore generally assumed that selection on standing genetic variation should dominate the dynamics of evolution experiments with sexual species. Thus, only soft-sweep dynamics can be evaluated using genomic data from existing experimental evolution studies of small sexual populations.

While several well-known experimental evolution systems that use sexual organisms exist (e.g. crickets, butterflies, mice; cf*.* [11] and references therein), the most common among them is *Drosophila*. To date, there is a small but growing number of *Drosophila* evolution experiments that probe whole-genome sequence data for evidence of selective sweeps. The following section is thus restricted to these few studies, but recently published work using two long-established vertebrate systems bears discussion. In seven independent mouse lines selected for high body weight, Chan *et al.* [23] were able to identify 67 regions where high lines shared variants rarely found among controls. Most of these regions are contained within intervals previously identified by quantitative trait locus (QTL) mapping, and some overlap loci associated with human height variation. The authors sequenced these regions in mice from a single selection experiment, and show evidence of extreme reductions of heterozygosity. This is fairly convincing evidence for single-origin soft sweeps in this system, and when mice from natural populations were sequenced at these locations, reduced heterozygosity was observed as well. Of course, maintenance issues render mouse systems impractical for addressing questions about the dynamics of adaptive alleles, but this work illustrates how evolution experiments can potentially shed light on the genetics of natural populations. Data from animal domestication studies are also useful in an experimental context, as for some breeds, lines that have been selected in opposite directions for a focal trait are available for study. The Virginia chicken is an example of such a breed; genomic sequencing of these lines found strong evidence of both complete and incomplete soft sweeps after both 40 and 50 generations of selection for increased or decreased body size [20]. The authors identify single nucleotide polymorphisms (SNPs) that are alternately fixed in the two populations, and cluster them by proximity; by this method, they find at least 50 of these clusters, which are signatures of complete soft sweeps. They also find pervasive differentiation between the populations that has not fixed, and interestingly in at least 10 of these regions, differentiation increased between 40 and 50 generations of selection. This highlights the importance of collecting sequence data from evolution experiments at multiple timepoints. Johansson *et al.* [20] find evidence that the tempo of adaptation from standing genetic variation differs depending on the variant, and future studies could aim to define the circumstances affecting this. Despite the operation of severe bottlenecks, high levels of inbreeding and intensive selection during the history of domestication, most domestic animal species are genetically diverse (reviewed in [37]), and thus may serve similar roles as laboratory-selected populations for studying the dynamics of adaptive alleles.

## 5. Experiments with *Drosophila* do not yield general results

**Figure 1.**
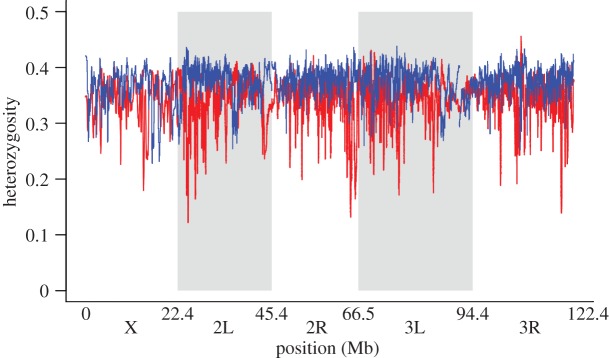
Heterozygosity throughout the genome in experimental populations described in Burke *et al.* [33]. Sliding-window analysis (100 kb) of heterozygosity in the control lines (blue), and those selected for accelerated development (red). Sliding-window size was chosen based on the estimated level of linkage disequilibrium in these populations, and averages were taken with a 2-kb step size. Genomic DNA libraries were prepared from a total of 125 female flies, pooled from five replicate populations for each treatment. The *x*-axis represents the five major chromosome arms of *D. melanogaster* in physical distance (Mb).

Burke *et al.* [14] describe the first genomic analysis of experimentally evolved, sexually reproducing populations. This study examined whole-genome sequence data from pooled samples of populations of *Drosophila* that had experienced over 600 generations (20 years of real time) of selection for accelerated development, as well as their ancestral control populations. This technique of analysing pooled samples allows for estimates of allele frequencies and heterozygosity in an entire population. The primary goal of this study was to identify SNPs with significantly different frequencies in the experimental and control populations, and excess of 20 000 such SNPs were identified. Burke *et al.* [14] also quantified heterozygosity at these SNPs, and found it reduced in the experimental populations relative to the controls. Although local losses in heterozygosity were observed in the same areas of the genome at which there was significant differentiation in allele frequencies, in no region did heterozygosity come close to zero, along a 100 kb sliding-window average (figure 1). In this study, experimental treatments all exist as fivefold replicate populations. The authors did not sequence genetic material from all of these replicate populations, but they did compare sequence data from one replicate population to data from the pooled replicates, as well as genotyped a number of individuals from all five replicates. These two approaches both supported the idea that selection operated on the same alleles in each replicate population. This evidence of parallel evolution can be interpreted as adaptation via standing genetic variation, and it is also suggestive that genetic drift alone did not drive allele frequency change. Importantly, these results cannot unequivocally distinguish between the soft-sweep patterns, but the authors speculate that the local losses in heterozygosity are incomplete, single-origin soft sweeps.

Turner *et al.* [21] recently described whole-genome resequencing data from populations of *Drosophila* selected for increased and decreased body size. For 100 generations, the most extreme 160 males and 160 females in each selected line were allowed to reproduce (approx. 10% of the individuals screened). Two replicate ‘high’, ‘low’ and ‘control’ treatments were all handled in parallel. Following the resequencing of pools of individuals from each replicate, genome-wide allele frequency divergence and heterozygosity were quantified. The authors identify at least hundreds, and likely thousands, of polymorphisms that affect body size in this long-term laboratory population. Turner *et al.* [21] reported reduced heterozygosity in the selected populations, especially in the ‘low’ reduced-size populations where the most phenotypic change occurred. While they found that variation persists across most of the genome, they do identify regions (approx. seventy 10-kb windows) with near-zero heterozygosity in each of the replicate ‘low’ populations. These sweep signatures appear to conflict with the observations of Burke *et al.* [14]; however, it is likely that important differences in experimental design explain this, including long-term effective population size being an order of magnitude smaller, and the strength of selection being presumably greater.

Zhou *et al.* [22] recently described genomic data from populations of *Drosophila* selected for hypoxia tolerance for over 200 generations, starting from an ancestral laboratory population composed of admixed isofemale strains. They find that two independent, hypoxia-tolerant replicate populations, reportedly maintained at long-term *N*_e_ ∼ 10^3^, had the same genomic intervals undergo a high degree of fixation; specifically, they identified 24 such regions containing a total of 188 genes in the experimental populations compared with controls. These regions were almost all found on the X chromosome, suggesting that genes required for adaptation to severe hypoxic conditions are localized rather than distributed across the genome. This study follows up the identification of these candidate regions with assays to confirm loss or gain of function in implicated genes, and the contribution of the Notch signalling pathway to hypoxia tolerance was confirmed using a suite of molecular biology techniques (Notch mutants, RNAi-mediated knockdown, pharmacological inhibitory reagents).

To what degree do these three *Drosophila* experiments present conflicting evidence of how adaptation proceeds in sexually reproducing populations? In all three, initially diverse ancestral populations achieved phenotypic and genotypic differentiation in a modest amount of time (less than 1000 generations) and with population sizes too small for de novo beneficial mutations to be common. Therefore, either drift or selection on standing genetic variation drives allele frequency change in each of these experiments. Burke *et al.* [14] find evidence of either incomplete soft sweeps or complete soft sweeps involving the fixation of variants harboured on haplotypes that are not identical by descent, but the data are not amenable to distinguishing between these two models. Turner *et al.* [21] and Zhou *et al.* [22] appear to find some evidence for complete soft sweeps. This could be partially explained by the different effective population sizes in the different experiments, as it is conceivable that when strong selection is applied to sufficiently small populations, drift effects will result in the complete fixation of standing variants. While this seems intuitive for the body size selected populations where *N*_e_ ∼ 10^2^, the other two experiments use populations of sizes that are an order of magnitude greater. This disparity could also be a result of the nature of the phenotypes under direct selection. Both Burke *et al.* [14] and Turner *et al.* [21] describe the extensive maintenance of genetic variation in their experimental populations, despite dramatic phenotypic changes. Zhou *et al.* [22], on the other hand, do not report observing this phenomenon, and instead focus on the relatively small number of genes where they find evidence of sweeps. It seems reasonable to suppose that hypoxia tolerance is a less polygenic trait than either development time or body size, where there is only one or a few adaptive solutions to achieve a phenotypic optimum. In addition, rather than starting with an already domesticated, previously outbred population, the hypoxia-tolerant populations were established by crossing individuals from 27 distinct isogenic strains. This suggests large, common haplotype blocks segregating in the early generations of selection, and a high probability that beneficial standing variants reside on haplotypes that are identical by descent. This experimental design therefore is likely biased towards the outcome of a small number of complete soft sweeps genome-wide.

## 6. Manipulating complex mating systems may result in better study of mutational dynamics

In organisms such as yeast or *Caenorhabditis elegans* with complex mating systems, it may be possible to combine the practical features of asexual evolution experiments with the ability to maintain standing genetic variation. Morran *et al.* [18] describe experiments where they manipulate *C. elegans* populations such that they either consist of obligate outcrossers or obligate self-fertilizers. This approach allowed them to directly compare the fitness of these populations following parallel selection treatments, and thus address questions about the evolution of outcrossing. The experiments did not begin with standing genetic variation, and the populations were not surveyed genomically (e.g. by whole-genome resequencing); however, such a system provides a promising solution to some of the limitations discussed to this point. Specifically, experiments could be designed with very large populations, harbouring initial standing genetic variation, and incorporating recombination such that both hard sweep and soft-sweep patterns could potentially be detected.

**Figure 2.**
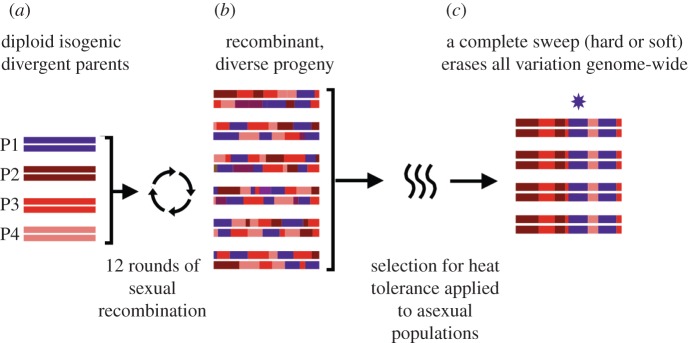
Schematic of experimental design used by Parts *et al.* [17]. (*a*) Four isogenic parental yeast strains were experimentally manipulated to induce several rounds of sexual recombination, generating a pool of recombinant progeny with millions of unique haplotype blocks segregating. (*b*) This pool was then subjected to selective pressure for heat tolerance, and the asexually reproducing yeast populations were monitored for changes in parental allele frequency. While a number of QTL regions were identified, the authors noticed ceases in allele frequency changes, and a dearth of fixation events, after 200 generations. (*c*) A visualization of diversity under a complete sweep scenario, which *was not observed* in this experiment. If selection were to act on a standing variant or a new mutation (at the region indicated by the star), the entire genetic background on which the variant resides would fix, eliminating all variation in the population.

In a similar vein, Parts *et al.* [19] recently describe an innovative approach for establishing a *S. cerevisiae* evolution experiment with abundant standing genetic variation, using outbred, recombinant diploid individuals. While their approach was designed for general use as a QTL mapping tool (cf. [38]), the strategy and resulting data have potential for distinguishing mutational dynamics. The strategy starts with a four-way cross between highly diverse parental strains, followed by multiple rounds of sexual recombination, resulting in several million highly recombined haplotypes (figure 2). Parts *et al.* [19] then applied laboratory natural selection to the resulting pools of yeast progeny, as they grew *asexually*. The pools were sequenced at various time points during the selection process, and when analysed for genome-wide allele frequency differences between control and selection treatments, no evidence for complete fixation was found. However, as only approximately 200 generations of selection had elapsed by the time of genomic analysis, this is likely not enough time for either standing variants with small selection coefficients to sweep, or de novo beneficial mutations to arise and sweep.

The experimental design of Parts *et al.* [19] subjects an assemblage of different clonal haplotypes generated by initial rounds of recombination to a selection treatment. Theoretically, this experimental design allows for both hard sweeps from new mutations *or* soft sweeps from the initial standing variation, but as soon as the first sweep occurs, standing genetic variation is wiped out. However, if recombination were to occur regularly over the course of selection, this would unlink adaptive mutations from other genomic variation and make future sweeps from standing variation detectable.

## 7. Conclusions and directions for future studies

Many outstanding questions remain about the origins and fates of adaptive mutations. Selective sweeps, as evidenced by footprints of reduced genomic variation, provide one method for identifying regions of the genome that have been important in adaptation in the recent past. Genome-wide scans of polymorphism in *Drosophila* provide convincing evidence that hard sweeps have been common throughout the evolutionary history of this model organism [39]. On the other hand, a recent study observes evidence of complete soft sweeps at genetic loci associated with pesticide resistance [9], and in the *Drosophila* evolution experiments described here, evidence for both incomplete and complete soft sweeps was found. These conflicting observations raise many questions about the nature of the adaptive process. Under what circumstances does natural selection act primarily on new advantageous mutations versus existing variation? Are these circumstances unifying across taxa? What are the roles of effective population size, strength of selection and number of generations in determining the complete fixation of alleles? As it allows investigators to manipulate each of these variables directly, laboratory natural selection can potentially test these questions.

There are commonalities to be synthesized from recent genomic analyses of both microbial and *Drosophila* selection experiments. The most startling of these is the observation of a high degree of genetic variation in evolving populations. In initially clonal microbial populations, mutations do not necessarily arise and fix in a simple step-wise manner; rather, multiple mutations arise, interact and interfere with each other's substitution in ways that defy theoretical predictions. In sexually recombining populations with initial standing variation, a surprising amount of that variation is maintained. In experimentally evolved *Drosophila* populations, heterozygosity is not always eliminated at regions putatively under selection.

So how can future work more directly characterize the dynamics of adaptive mutations? For one, the existence of independent evolutionary replicates is essential for the ability to distinguish hard sweeps from soft sweeps in an evolution experiment with recombination (table 1). Observing a sweep at the same genomic location involving the same ancestral haplotype in independent replicate populations suggests that the sweep results from standing genetic variation, and the more times this phenomenon is observed, the more statistical power exists to support the case. A recent exceptionally well-replicated microbial study suggests that even the existence of over 100 independent replicates may not be sufficient to fully characterize the diversity of adaptive strategies employed by populations under a selective pressure [13]. The number of independent replicate populations required to adequately distinguish among sweep patterns may thus be quite high, but evolution experiments in general tend not to be very highly replicated, and *Drosophila* evolution experiments in particular achieve a maximum of three- to fivefold replication [40].

The development of laboratory systems such as those described in [18,19] is very promising for the experimental study of adaptation. These systems feature populations of organisms with short generation times that can be handled in populations large enough such that de novo beneficial mutations are likely to arise during the course of an experiment. These systems have been manipulated such that either asexual or sexual reproduction may occur, and the investigator can control this variable. In the case of Parts *et al*. [19], yeast biology prohibits sexual recombination to take place every distinct generation, but populations of individual cells could be synchronized and forced to undergo meiosis at planned times. Thus, in these systems, if adaptive alleles become fixed, standing genetic variation may still be maintained. This creates opportunities for experiments in which population size, selective regime and length of selection can all be tuned, and adaptation from either new mutations or standing genetic variation can be monitored. For these future evolution experiments to optimally bear on questions about the mechanisms of adaptation, it is of paramount importance that they are carried out with as much replication as possible, and as much genomic sampling as possible. As genomic technologies continue to improve and are growing more cost-effective, this is rapidly becoming a more realistic goal.
